# An Overview of the Development and Preclinical Evaluation of Antibody–Drug Conjugates for Non-Oncological Applications

**DOI:** 10.3390/pharmaceutics15071807

**Published:** 2023-06-24

**Authors:** Lal Bahadur Pal, Prajakta Bule, Wahid Khan, Naveen Chella

**Affiliations:** 1 Department of Pharmaceutical Technology (Formulations), National Institute of Pharmaceutical Education and Research (NIPER), Guwahati 781101, Assam, Indiabuleprajakta2@gmail.com (P.B.); 2 Natco Research Centre, Natco Pharma Ltd., Hyderabad 500018, Telangana, India

**Keywords:** inflammation, immunology, pharmacokinetics, clinical trial, safety, therapeutic efficacy

## Abstract

Typically, antibody–drug conjugates (ADCs) are made up of a humanized antibody and a small-molecule medication connected by a chemical linker. ADCs’ ability to deliver cytotoxic agents to the specific site with reduced side effects showed promising results in oncology. To date, fourteen ADCs have been approved by the US Food and Drug Administration, and approximately 297 ADCs are in pre-clinical/clinical stages in the oncology area. Inspired by these outcomes, a few scientists explored the potential of antibody–drug conjugates in non-oncological conditions such as arthritis, myasthenia gravis, immunological disorders, and kidney failure. However, there are limited data available on the non-oncological applications of antibody–drug conjugates. This current review focuses on the non-oncological applications of antibody–drug conjugates, their developmental studies, testing procedures, in vitro evaluations, and pre-clinical testing. Additionally, a summary of the restrictions, difficulties, and prospects for ADCs in non-oncological applications is provided.

## 1. Introduction

**Figure 1:**
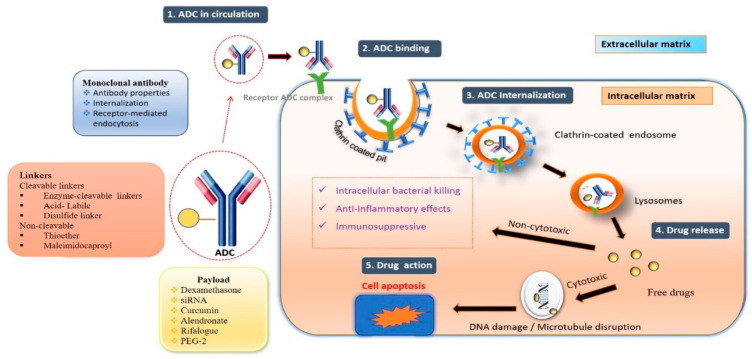
Structure and mechanism of action of ADC.

Antibody–drug conjugates (ADCs) are frequently used to treat cancer and are composed of a monoclonal antibody (mAb) connected to a cytotoxic agent through a suitable linker. They combine the finest parts of monoclonal antibodies with small molecules to create a highly selective and effective moiety for the target site. ADCs, called “homing missiles”, have three essential components: a mAb that preferentially binds to an antigen surface of the target tissue, an active therapeutic molecule, and a linker (either cleavable or non-cleavable) [1]. The most common carriers for ADCs are monoclonal antibodies, which have strong binding and target selectivity [2]. The active moiety may be a cytotoxic agent in the case of cancer or other small molecules in the case of non-oncological applications, as shown in Figure 1. The significant advantage of ADCs is the reduction in off-target toxic effects, which makes them expedient in oncology for delivering a cytotoxic payload to a particular site. The application of ADCs in oncology started with the approval of Mylotarg^®^ (Gemtuzumab ozogamicin) by the United States Food and Drug Administration (FDA) for CD33+ acute myeloid leukemia. Pfizer removed Mylotarg^®^ from the US market in 2010 in response to a request from the US FDA based on a clinical trial that revealed safety concerns and no clinical advantages for patients. However, the Japanese Pharmaceuticals and Medical Devices Agency (PMDA) decided that there were no major changes in the safety profile and that the product could be marketed in Japan [3,4,5,6,7]. Later, in 2017, the FDA reapproved Mylotarg^®^ for a different population with a modified dosage regimen (three doses of 3 mg/m^2^ instead of one dose of 9 mg/m^2^ previously) that lowered the maximum plasma concentration and enhanced safety [8]. Adcetris^®^ (brentuximab vedotin), comprising a CD30-specific mAb conjugated to monomethyl auristatin E (MMAE), is the second ADC to reach the oncology market after approval by the US FDA in 2011. In the United States, Europe, and Japan, it is authorized for Hodgkin lymphoma (HL) and systemic anaplastic large cell lymphoma (sALCL). With the binding of MMAE to tubulin, Adcetris^®^ disrupts the cell’s microtubule network, resulting in cell cycle arrest and apoptotic cell death [9,10,11].

Kadcyla^®^ (ado-trastuzumab emtansine), the first ADC approved for treating solid tumors, was developed by Genentech/Roche in 2013. It comprises the anti-HER2 IgG1 antibody trastuzumab, linked through a non-cleavable thioether linker to the antimitotic agent, DM1 (a maytansine derivative). It causes cell cycle arrest and apoptotic cell death by disrupting the microtubule network. It is approved as an adjuvant (after surgery) therapy for patients with HER2+ early breast cancer who have previously undergone trastuzumab (Herceptin^®^) and a taxane, either alone or in combination. This approval marked a turning point in ADC research since treating solid tumors with such therapy has been daunting. [12,13,14]. These ADCs gained more attention in the oncology area due to their success story.

Currently, fourteen ADCs have already been approved worldwide for solid tumors and hematological malignancies, and many more ADCs have moved through different stages of clinical development [15]. When the term antibody–drug conjugate was searched on clinicaltrials.gov (as of 19 June 2023), 235 clinical trial studies were found. Approximately 122 studies were active and recruiting at the time, 65 were finished, 7 were withdrawn for various reasons, and 28 were terminated. The vast majority of them are cancer-related, with DNA or microtubule-damaging chemicals as payload [16].

Although ADCs have shown outstanding potential in oncology, their application beyond cancer has been minimal but growing in recent years. The idea of employing E-selectin-directed immunoconjugates to deliver dexamethasone to activated endothelial cells by Everts et al. [17] was the beginning of the non-oncological applications of ADCs. Since then, there have been a few attempts to explore the utility of ADCs in various non-oncological conditions, including inflammatory disorders (arthritis, atherosclerosis, muscular disease, myasthenia gravis, systemic sclerosis, and ulcerative colitis), bacterial infections, cardiovascular disease, and kidney failure. The majority of these studies examined the potential of ADCs in vitro cell lines or animals [18]. Table 1 gives an overview of different ADCs that are in various stages of preclinical and clinical states with an emphasis on non-oncology.

**Antibody–drug conjugate mechanism of action:**
The fundamental mechanism after the entry of ADCs into the body is discussed below [38].
**Circulation:** Antibody–drug solution administered by intravenous route enters the bloodstream.
**Binding:** The mAb component of the ADC binds to the target antigen.
**Internalization:** Internalization of ADC occurs via receptor-mediated endocytosis.
**Recycling:** Early endosomes’ fraction of ADC binds to FcRn receptor.
**Release:** Lysosomes fuse with late endosomes and release active moiety.
**Action:** The cytotoxic drug interacts with DNA/microcellular assembly and induces apoptosis [39] in cancer or shows its reported activity in other cases, as shown in Figure 1.

## 2. ADCs beyond the Cancer Treatment

The substantial increase in ADCs has been seen in non-oncological applications, where the payload can change specific biological processes instead of directly killing the cells. For example, antibiotics as a payload can be used to treat bacterial infections, and glucocorticoids can be used to suppress the immune system.

### 2.1. Anti-Inflammatory

Inflammation is a protective mechanism of the body characterized by triggering immune and non-immune cells that safeguard the host from various infections and promote tissue restoration and healing [40]. However, chronic inflammation due to altered body conditions leads to many conditions, such as hypertension, cardiovascular diseases, metabolic diseases, arthritis, psoriasis, and cancer. Glucocorticoids are among the many anti-inflammatory agents available in the market prescribed to treat inflammatory conditions such as asthma, autoimmune disorders, arthritis, and inflammatory bowel disease. However, due to their off-target exposure, the chronic use of steroids causes severe side effects [41]. Few researchers evaluated the potential of ADCs to deliver these corticosteroids with minimal side effects. Even though the major focus of the present discussion was on the site-specific delivery of glucocorticoids, the delivery of natural bioactive like curcumin was also discussed below.

#### 2.1.1. Anti-E Selectin Dexamethasone Conjugates

Everts et al. [17] evaluated the ability of ADCs to deliver dexamethasone (Dex) intracellularly into the activated endothelial cells by exploiting E-selectin-directed immunoconjugate. E-Selectin (CD62E) is expressed in endothelial cells and is upregulated by various pro-inflammatory chemicals, such as Tumor Necrosis Factor (TNF), Interleukin-1 (IL-1), and LPS (lipopolysaccharide), via transcriptional regulation. So, an anti-E-selectin antibody (Eab) (H18/7) that can bind specifically to the over-expressed E-selectin was chosen. Dex was covalently linked with the antibody (H18/7) by a succinate linker. Sodium Dodecyl Sulfate-Polyacrylamide Gel Electrophoresis (SDS-PGE) and high-performance liquid chromatography (HPLC) were used for the qualitative and quantitative analysis of conjugation, respectively. SDS-PGE analysis followed by Western blotting confirmed the conjugation of Dex with Eab, and conjugation efficiency was found to be 56% with DAR 2.3 (2.3 Dex molecules per antibody). Further, they also found that increasing the Dex concentration reduces the solubility of conjugate and the ability to target E-selectin. The sensograms obtained using surface plasmon-binding technology indicated the binding of the Dex–Eab conjugate to the E-selectin. The endothelial cell binding analysis of Dex ADCs was assessed by immunohistochemistry and flow cytometry in human umbilical vein endothelial cells (HUVEC). Immunohistochemical analysis of mAb and the conjugated Dex showed accurate binding of the conjugate to rhTNF-stimulated HUVEC. The conjugated Dex binds to activated endothelial cells but not to quiescent endothelial cells, as confirmed by Flow cytometry. Conjugated and unconjugated antibodies are similarly selectively bound to activated HUVEC.

Following binding, Dex should be released from the ADCs to produce the required pharmacological action. Confocal laser scanning (CLMS) was used to determine the internalization and binding of Dex antibody conjugates using HUVEC stimulated with rhTNF-alpha. The internalization of conjugated and unconjugated antibodies by endothelial cells was identified based on staining patterns. The visibility of staining on the surface during the initial 10–20 min, followed by increased intracellular intensity after one hour, indicates initial binding on the surface and the internalization of the conjugate later. The decline in intracellular intensity after 18–24 h of incubation suggests the breakdown of the conjugate and is confirmed by immuno-transmission electron microscopy analysis. The release of Dex from the conjugate was studied by correlating the Dex concentration to the increase in the luciferase reporter gene. The results confirmed that Dex–Eab conjugates release the drug at a slower rate to the cells than unconjugated Dex. Finally, the effect of released Dex on the pro-inflammatory IL-8 mRNA levels was estimated by Western blotting. The decrease in IL-8 expression levels confirmed that Dex released from the conjugate was active and shows the intended pharmacological activity. Based on the results obtained, the authors concluded that the synthesized conjugate has the potential to deliver Dex specifically into activated endothelial cells. However, further in vivo studies must be performed to confirm the same.

#### 2.1.2. Anti-CD163 Dexamethasone Conjugates

Glucocorticosteroids act as anti-inflammatory agents by suppressing the release of pro-inflammatory and tissue necrosis factors from the macrophages. However, the use of these agents is limited by severe side effects, such as muscle mass loss, bone mobilization, immunosuppression, and metabolic alterations due to the non-specific localization of glucocorticoids. Many inflammatory diseases, such as atherosclerosis, rheumatoid arthritis, and inflammatory bowel disease, are characterized by elevated hemoglobin scavenger receptor CD163 on activated macrophages. Graversen et al. [19] demonstrated the production and characterization of ADC for the targeted delivery of glucocorticoids against inflammation. ADCs were synthesized by coupling dexamethasone (Dex) with mouse anti-rat CD163 mAb. The anti-inflammatory potential was tested in vitro and in vivo using lipopolysaccharide induced inflammation. Anti-CD163 mAb–Dex conjugate is the first non-oncological antibody–drug conjugate for inflammatory disorders. 

The reaction between the primary amino groups of the anti-CD163 and Dex–hemisuccinate–NHS esters results in the formation of anti-CD163–Dex conjugate. The final formulation consists of four Dex molecules per antibody with less than one percent of free Dex. Structural integrity, reactivity, and specificity are the primary characteristics of the antibody that should be retained after conjugation to maintain its potential. Gel electrophoresis and size exclusion chromatography confirm the integrity by noting the absence of any aggregation. The reactivity and specificity of the antibody after conjugation was confirmed by Western blotting and flow cytometry analysis using Chinese hamster ovary (CHO) cells expressing rat recombinant CD163. The use of 125I-labeled anti-CD163 revealed that only transfected cells can uptake the ADCs compared to non-transfected cells, which is an indication of specificity. The increase in Free 125I in CHO after 2 h indicates the liberation of the drug from the conjugate. This was further confirmed by partial colocalization of antibody and dexamethasone in CHO cells after two hours, as observed under confocal microscopy. 

The anti-inflammatory effect was evaluated by measuring LPS-mediated TNF-alpha formation in the rat macrophages after incubating anti-CD163 dexamethasone, free Dex, IgG–Dex (control conjugate), anti-CD163, and buffer for 15 min. Except for anti-CD163 and vehicle control, all three other conjugates showed reduced TNF-α production via the inhibition of LPS-mediated stimulation of rat macrophages. It was found that anti-CD163 Dex is more effective than free Dex during the initial 15 min, and after 1 h, no difference was observed. This was explained by the slow passive diffusion of free Dex compared to the endocytosis of conjugate. 

The in vivo anti-inflammatory activity of the developed ADC was evaluated in LPS-injected rats. After induction of inflammation, anti-CD163 Dex (ADC) and free Dex were injected into rats, and serum TNF-α levels were measured to assess the anti-inflammatory activity. In comparison to Dex, anti-CD163–Dex showed superior efficacy. Non-conjugated anti-CD163 showed the same elimination half-life as anti-CD163–Dex at the exact dosage. The absence of significant weight reduction of the thymus, spleen, and total body of rats that received ADC compared to those that received free Dex confirms the site-specific delivery of conjugated drugs from ADCs.

#### 2.1.3. Antibody–Curcumin Conjugates Anti-DR5

Liver fibrosis is characterized by severe liver damage and the accumulation of extracellular matrix proteins. The regeneration of damaged hepatic tissue is linked to inflammation-related cellular damage. Hepatic fibro-genesis is primarily attributed to activated hepatic stellate cells (HSCs) that raise the expression of death receptor 5 (DR5). Pro-inflammatory mediators activate HSCs, which promote macrophages toward pro-fibrotic phenotype. This triggers activated HSCs to transdifferentiate in a way that increases collagen deposition [42]. Curcumin is a natural phenolic antioxidant compound with anti-inflammatory and anti-fibrotic potential due to the deactivation of the HSCs [43]. You et al. [44] developed anti-DR5 antibody conjugates containing curcumin to treat liver fibrosis and enhance the clearance of HSCs. Curcumin ADC was synthesized through a four-step reaction wherein curcumin was modified to include maleimidohexanoic acid in the first three steps. In the final step, the maleimide group of the curcumin was coupled to the thiol group of the anti-DR5 antibody. After preparation, each compound was characterized by 1H-NMR mass spectrometry. The curcumin-to-antibody ratio was found to be 3:1 in the ADC as measured by a Nano-drop spectrophotometer. The Coomassie blue protein analysis confirmed the stability of the synthesized conjugate as there was no significant change in the weight of the conjugate after incubation for 48 h in phosphate buffer. Comparative analysis of Flamma^®^ 675-labeled bare antibody and antibody–curcumin conjugate uptake under confocal microscopy revealed no significant difference in the uptake of conjugate and bare antibodies, indicating that the conjugation did not alter the binding characteristics of the antibody. 

Reduction in the conjugate uptake was observed in the LX-2 activated cells in the presence of free anti-DR5 antibodies compared to activated LX-2 cells without antibodies. Additionally, conjugate uptake was significantly higher in LX-2-activated cells compared to the HepG2 and RAW264.7 cells. This indicates that the uptake was due to the overexpression of DR5 in activated HSCs. MTT (3-[4,5-dimethylthiazol-2-yl]-2,5 diphenyl tetrazolium bromide) assay findings demonstrated bare antibodies and their curcumin conjugates were toxic on the activated LX-2 cells but not to HepG2 and inactivated LX-2 cells. This confirms that the induction of apoptosis by antibody–curcumin conjugate occurs only in the activated HSCs. 

The antioxidant and anti-inflammatory activity of antibody–curcumin conjugate was determined by the reactive oxygen species (ROS) levels and inducible nitric oxide synthase (iNOS) expression in activated LX-2 cells, respectively. ROS and iNOS were produced by activated HSCs [45]. Transforming growth factor beta (TGF-β1), in particular, stimulates LX-2 cells, increasing their ROS levels inside the cell [46]. Antibody–curcumin conjugate reduced the ROS levels significantly compared to pure curcumin in LX-2 cells, as observed under confocal microscopy, indicating a higher antioxidant effect. Similar results were also observed in the case of iNOS levels, further confirming the effectiveness of synthesized antibody–curcumin conjugate in the activated HSCs compared to nascent HSCs. The anti-fibrotic potential of antibody–curcumin conjugate was appraised by measuring the alpha-SMA level in activated LX-2 cells using immunocytochemical analysis and flow cytometry. The result concluded that antibody–curcumin conjugate showed significant enhancement in the anti-fibrotic effect compared to pure curcumin and bare antibodies, confirming the synergistic activity of the antibody and curcumin combination in the synthesized conjugate. 

The bio-distribution and pharmacological activities of the curcumin from the antibody conjugate were evaluated in mice. After injection of Flamma^®^ 675-labeled antibody–curcumin conjugate to normal and liver fibrosis-induced animals, conjugate distribution was higher in the fibrosis liver than in the normal liver, confirming the targetability observed in vitro. 

### 2.2. Antibody-Mediated siRNA Conjugates

**Figure 2:**
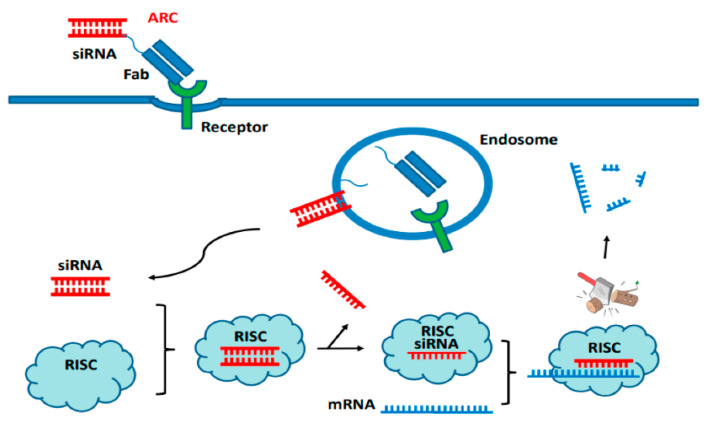
The mechanism of action of antibodies conjugated with short interfering RNA (siRNA) Adapted from [47].

Small interfering RNA (siRNA)-based therapy has gained much curiosity from researchers due to its potency. However, concerns like adverse effects due to off-target exposure limit their application. Noncovalent hybridization or the direct oligonucleotide conjugation of siRNA to the antibodies or their fragments (Fab, scFv) generates the antibody–siRNA conjugates (ARCs). The antibody–siRNA conjugates (ARCs) show specific transport into tissues, cells infected with viruses, or cancer cells, both in vitro as well as in vivo. Cell surface receptors bind ARCs carrying Fab and siRNA, which are then taken up by endosomes and transported into the cell (Figure 2), where the siRNA is released and escapes into the cytoplasm. After the siRNA has been loaded into RISC (RNA-induced silencing complex), specific mRNA is degraded. This concept is utilized when nucleic acid is used as a payload [47].

ADCs, due to their site-specific delivery, attracted the researchers to combine the antibodies with siRNA for an efficient therapeutic potential. In their recent article, Cao W et al. [48] discussed the different approaches for conjugating siRNA with antibodies. They explained various conjugation mechanisms and different linkers used to conjugate the siRNA with antibodies. The authors also mentioned a few examples of ADCs and their application in multiple diseases. A few of them are discussed below.

#### 2.2.1. Myasthenia Gravis (MG)

Myasthenia gravis (MG) is a rare autoimmune disease that causes muscle weakness due to the destruction of acetylcholine receptors (AChR) at the neuromuscular junction by autoantibodies. Among all MG patients, about ninety percent will have anti-AChR auto-antibody in their blood [49]. The most common ways of treating MG are using steroids, immunosuppressive therapies, and plasmapheresis. These treatments have many side effects, such as lowering the immune system, causing infections, and raising blood pressure. So, there is an emergent need for new and better ways to treat MG. The autoimmune disease MG depends on T cells and is mediated by B cells. The receptors responsible for mediating the autoimmune manifestation of MG were found in the B cells [50].

The survival of mature B cells is maintained by nuclear factor kappa B (NF-kB)-mediated cell signaling caused by the B-cell-activating factor (BAFF) receptor, primarily expressed in mature B cells. Apart from B cell maintenance, the pathogenesis of MG can be interrupted by silencing the specific gene expression present only in activated B cells. The bispecific, i.e., targeting particular receptors and gene silencing, may be an excellent therapeutic option for MG. Ibtehaj et al. [24] tried to solve the principal problem of MG by using an antigen–antibody complex. They investigated the therapeutic effects of BAFF receptor-specific mAb conjugated with short interference RNA in a myasthenia gravis (EAMG) mice model. First, they allied the protamine linker to the antibody for conjugation using Di-Methyl Formamide in a conjugation buffer. The resultant antibody with linker conjugated with duplex siRNA (~19 nucleotides) (1:1) by the electrostatic reaction. Electrophoresis was used to confirm the effectiveness of conjugate production and conjugate stability.

After BAFF receptor-specific conjugation, the authors tested the internalization ability of the conjugate into the cells by transfecting Cy-3 tagged non-specific siRNA in a rat myeloma Y3 cell line. According to the findings, about 80% of Y3 and 70% of B cells fluoresced red. The efficacy of the conjugate to reduce BAFF receptor expression in mature B cells was estimated in rat myeloma Y3 B cells and the C57BL/6 EAMG mice model. The Y3 cells with BAFF receptor-specific conjugate or siRNA-, IgG-, or IgG–siRNA-controls were incubated for 48 h. The Flow cytometry data showed that the conjugates downregulate the expression of BAFF receptors by about 50% compared to other controls. After the in vitro confirmation, they further assessed the inhibitory effect of the conjugate in vivo. For in vivo efficacy, the conjugates were given intraperitoneally (i.p.) to EAMG mice only once in three doses. The following doses were used: high dose, 350 g of each mAb and siRNA; low dose A, 125 g of each mAb and siRNA; and low dose B, 50 g of each conjugate component. The conjugate reduced a greater number of mature B cells (CD268+CD19+) than untreated or bovine IgG controls in peripheral blood mononuclear cells (PBMCs) at either higher dose or lower dose A. To better understand how BAFF receptor conjugate affects B cell phenotypes and survival in the spleen and lymph nodes, the authors examined how much Fas (CD95) was expressed in these cells. This protein was expressed in germinal center B cells only after cell death was triggered. Surprisingly, they found a lot of Fas-expressing CD19+ and B220+ cells in the lymph nodes but not in the spleens of EAMG mice that were only given the high-dose conjugate. 

The BAFF receptor conjugate-treated mice grip strength was measured digitally (using a Dynamometer) every three, four, seven, and nine weeks. Notably, compared to mice in other treatment/control groups, these mice had much stronger muscles. The finding shows that BAFF receptor conjugate is an excellent way to make EAMG mice’s muscles stronger. Further, they checked the Type I interferon gamma (IFN) and serum cytokine levels expression in lymphoid and PBMC cells in mice. They noted that the high-dose conjugate group showed a high level of IFN.

The study revealed that the BAFF receptor-specific mAb–siRNA conjugate could induce apoptotic signaling via Fas-dependent and -independent mechanisms in MG. Further improvements in the dose and formulation approaches may help to achieve a better outcome.

#### 2.2.2. Antibody–siRNA Conjugate in Muscular Disorder

Antibody–siRNA conjugate targeted to skeletal and cardiac muscles is the novel approach for treating muscular disorders. Sugo et al. [23] demonstrated the potential of anti-CD71 Fab-conjugated siRNA in silencing the effects in muscular organs via hypoxanthine-guanine phosphoribosyl transferase (HPRT). The antibody and siRNA were covalently linked via maleimide linker by Michael reaction, and HPLC and UV absorption spectral analysis confirmed the conjugation. An immunosorbent-binding experiment was performed to assess the specificity and binding activity of anti-CD71 siHPRT using recombinant mouse CD71 proteins. The study showed a concentration-dependent binding to CD71 proteins at nanomolar concentrations. 

Anti-CD71 siHPRT linked with the maleimide linker showed three-fold higher silencing efficiency than the IgG–Fab-conjugated siHPRT in the in vitro experiments using primary hepatocytes. This result suggests the specificity of the antibody selected for the target organs. Mice were administered anti-CD71 siHRPT (10 mg/kg) i.v dose, and significant silencing of the HPRT in the heart and calf muscles was observed post-24 h dosing. This silencing increased gradually with time, and maximum activity was observed after three days of injection. The absence of silencing after IgG siHPRT indicates that this effect was specific to the CD71.

Further, to study the effect of the administration route on the silencing ability of the conjugate, anti-CD71 siHPRT was administered through subcutaneous and intraperitoneal routes, and the silencing efficacy was compared with that of intravenous administration. Based on the results, the authors suggested that the conjugate can reach the target from any administration site. With the successful results of these experiments, the authors further explored the efficiency of anti-CD71–Fab conjugated to myostatin (anti-CD71-siMSTN) in mice models of peripheral artery disease. The results also indicated the silencing of myostatin mRNA. Based on the results, the authors concluded that using ADCs may act as a platform to deliver the siRNA for better efficacy and activity. 

### 2.3. Antibody–Antibiotic Conjugate

Antibiotics are often regarded as the hallmark of contemporary medicine. For decades, they have aided in preventing infectious disease epidemics and allowed for the safe practice of invasive surgery. The emergence of multi-drug resistance, along with a decline in the discovery and development of new antibiotics, has resulted in a global crisis with potentially disastrous repercussions. The application of ADCs in the treatment of challenging diseases such as cancer and autoimmune disorders has been very successful. A few researchers experimented with a similar concept and developed antibody–antibiotic conjugates (AACs), which combine the benefits of antibodies and antibiotics into a hybrid structure to address the limitations associated with antibiotic resistance [51,52]. MRSA (Methicillin-resistant *S. aureus*), resistant to all beta-lactam antibiotics, has emerged and expanded rapidly over the last few decades, making *S. aureus* infections more challenging to treat [53]. Lehar et al. [29] demonstrated that intracellular MRSA is difficult to remove with standard antibiotics, including vancomycin, linezolid, and daptomycin, used against invasive MRSA infections. *S. aureus* can invade phagocytic cells from there and also invades non-phagocytic cells, and this intracellular *S. aureus* is responsible for many ailments. It is necessary to clear intracellular MRSA to improve clinical success. 

Lehar et al. developed antibody–antibiotic conjugates (AAC) that are triggered precisely in mammalian cells, which are reserved by *S. aureus*. This AAC comprises an anti-*S. aureus* antibody (THIOMAB) that is covalently linked to a most effective antibiotic via a cathepsin-cleavable linker with a new quaternary ammonium salt. The prepared AAC does not have any antibacterial effects when coupled to *S. aureus* planktonic and does not enter mammalian cells due to the larger antibody size. However, after the consumption of AAC-opsonized bacteria by the host cells, the cleavable linker detaches and releases the antibiotic. Depending on the selectivity of the antibody, as many AACs as possible will be attached to bacteria to present sufficient antibiotic concentration at the required site of action [54]. The authors reported higher binding for the wall-teichoic acids (WTAs) after screening around 40 *S. aureus* antibodies obtained from the B cells of patients recovered from *S. aureus* infection. WTA produced by *S. aureus* is made up of repeating units of phospho-ribitol that may be converted into alpha or beta-O-linked N-acetylglucosamine (GlcNAc) TarM or TarS glycosyltransferases mediated by sugar. Rifalogue, a rifamycin derivative, was used as an antibiotic for conjugation with the antibody via tertiary amine. A fluorescence resonance energy transfer (FRET)-based probe was used to identify the linker cleavage using the same antibody (anti-beta WTA monoclonal antibody) conjugated to the dye. Further mass spectrometry also confirmed the separation of the linker from the cleavable linker in comparison to a non-cleavable linker obtained by replacing the amino acid at the P1 position. 

The AAC effectively opsonized MRSA, while the same bacteria opsonized with non-cleavable β-WTA antibody–antibiotic conjugates or mAb alone survived. Further, the effectiveness of the AAC to stop the cell-to-cell transfer of bacteria after intracellular targeting was 10-fold higher when compared to unconjugated rifampicin. The results indicated that AACs significantly reduced the viability of bacteria compared to the unconjugated antibiotic. 

Mice infected with intracellular MRSA were used to assess the efficacy of AAC in vivo. Infected mice were administered with phosphate buffer, vancomycin alone, or one dose of AAC. The bacteria that are resistant to vancomycin therapy can be successfully eradicated with a single dosage of AAC, indicating that AAC can eliminate the bacteria that escaped from vancomycin. The authors also found that treatment with AAC was more effective than unconjugated antibiotics, and the antimicrobial’s release from the AAC was crucial for effectiveness. This result indicates the possibility of antibody–drug conjugates for use outside of oncology [55]. 

Peck et al. [56] evaluated the safety, tolerability, pharmacokinetics, and immunogenicity of AAC in a phase 1 single-ascending dose study. The AAC (DSTA4637S) used in the study is composed of a new antibiotic, 4-dimethylaminopiperidino-hydroxybenzoxazino rifamycin (dmDNA31), and a human immunoglobulin G1 (IgG1) anti-*S. aureus* monoclonal antibody linked through a protease-cleavable valine-citrulline (VC) linker. The average DAR was found to be 2. The chosen mAb has selective binding to the bacterial wall and does not have any targeted antigen in human tissues, making the AAC safe to use. A total of 30 subjects were enrolled after screening the 104 healthy volunteers. The average age of the subjects was 41.9 years, and the ratio of male to female subjects was (63.3%: 33.3%). The subjects were treated with the AAC in four doses (5, 15, 50, 100, and 150 mg/kg, with n = 4 in each group) and placebo (n = 10). Except for treatment-emergent adverse events (TEAEs), there were no fatalities, adverse events, or severe side effects during the study. Both the placebo and the AAC-treated groups showed these TEAEs, with a high percentile (85%) in the AAC groups. In AAC-treated groups, there are also higher dose groups like 100 mg/kg (16 TEAEs) and 150 mg/kg (8 TEAEs). All the TEAEs are reversible and tolerable. No withdrawals were reported because of these TEAEs. A pharmacokinetics profile comparison made between AACs, pure antibodies, and unconjugated antibiotics indicated higher C_max_, lower protein binding, and better pharmacokinetics. The authors concluded that AAC showed future scope to be a novel delivery system against *S. aureus* infections with better tolerability, safety, and pharmacokinetics.

The capacity of antibody–antibiotic conjugates to deliver antibiotics with undesirable pharmacokinetics or toxicity profiles was proven by Kajihara et al. [57]. One of the difficulties in treating infectious diseases is the multidrug resistance (MDR) of bacteria, which raises mortality. *Pseudomonas aeruginosa* is a gram-negative bacterium responsible for some pulmonary and bloodstream-related infections. The development of resistance to these bacteria increased mortality and posed a critical problem. The MDR *P. aeruginosa* infection was recently designated as a “critical threat” pathogen by the World Health Organization. The authors selected mAb 26F8 as mAb with higher specificity and binding towards *P. aeruginosa* after the preliminary analysis. Later, AAC was generated by conjugating the antibiotic (G2637) to the mAb modified with six unpaired cysteines. In the presence of lysosomal cathepsins, the linker cyclobutane-1,1-dicarboxamide citrulline breaks down to release the attached antibiotic. LC-MS/MS analysis showed a DAR of 6 for the generated AAC. The cleavage of the linker and release of antibiotic was analyzed after incubating the AAC with *P. aeruginosa* in the presence and absence of cathepsin B. The presence of cathepsin B improved the level of inhibition compared to samples lacking cathepsin B, demonstrating that antibiotic release is a prerequisite for the AAC to function. This was further confirmed by quantification of the free antibiotic after adding the incubated AAC with *P. aeruginosa* PA14 WT to RAW264.7 macrophages. Here also, AAC containing cleavable linkers showed higher intracellular antibiotic concentrations in the macrophages over the AAC with non-cleavable linkers. The use of AAC indicated that the concentration of antibiotic required will be two-fold less compared to free antibiotics, indicating the efficiency of AAC. The authors came to the conclusion that using AAC allowed for larger intracellular antibiotic concentrations, which resulted in greater antibiotic efficacy as compared to using the free medication.

### 2.4. Glomerular Nephritis

Current treatments must improve specificity and reduce systemic toxicity to slow the progression of kidney disease. Kvirkvelia et al. [58] hypothesized that a human monoclonal antibody (F1.1) against the glomerular-localized noncollagenous-1 domain (NC1) of 3(IV) collagen may play the role of a carrier for precise drug administration. 3(IV)NC1 is a perfect target for the administration of disease-modifying medicines because of its augmented exposure during glomerular disorders and its subdued epitope expression in other organs.

The purified human monoclonal antibody was connected with the PGE2 or Dex through an amide bond formed between the carboxylic group of Dex or PGE2 and the mAb amide group using EDC (1-ethyl-3- [3-dimethylamino -propyl] carbodiimide hydrochloride) zero-length cross-linker. PGE2 and Dex were chosen because of their activity in nephrotoxic nephritis and anti-inflammatory properties, respectively. Using an ELISA kit for PGE2, they determined the amount of PGE2 bound to the antibody. Conjugate functionality was evaluated by measuring their ability to bind to podocytes using flow cytometry. The observation of low binding confirmed the antigenic specificity of the conjugates in additional cell lines (such as mouse mesangial cells and hepatocyte AML-12 cells). It inhibited the podocyte association by recombinant 3(IV). 

Animal studies were performed to identify the specificity and effectiveness of prepared ADCs in nephritis-induced animals. Nephritis was induced by injecting 13.5 microliters per gram of body weight via the intraperitoneal route. PEG2–F1.1, Dex–F1.1, and human IgG–Dex were administered at a 10 µg/g equivalent dose to the diseased animals after day 2. The efficacy was determined by measuring the protein in urine and blood urea nitrogen periodically. After 7 days of treatment, the kidneys were removed and examined by light microscopy or direct immunofluorescence. Strong and precise fluorescent staining was found inside the glomeruli of mice receiving the conjugate, allowing the conjugates to be easily identified within the glomeruli of diseased mice. It was confirmed that F1.1 conjugates mainly localized into the kidney since human IgG-reactive bands were individually seen in kidney lysates and not in lysates from other organs like the heart and lungs. A significant reduction in disease progression with normalized blood urea level (BUN) and histology was observed after treating with conjugates compared to the NTN mice that did not receive the conjugates. These results suggested that combining PGE2, which reverses existing nephritis, and a steroid (Dex), which reduces inflammation locally and throughout the body, potentially improves the recovery from preexisting nephritis at considerably lower doses when combined with human 3(IV) mAb. Anti-glomerular basement membrane antibodies target relevant epitopes since the expression is restricted to the kidney, a few other organs, and regions other than the glomeruli.

### 2.5. Rheumatoid Arthritis (RA)

Synovial membrane inflammation and bone degradation are the characteristics of rheumatoid arthritis, a severe chronic inflammatory disorder. Autoimmunity and tissue damage in RA are brought by excess pro-inflammatory cytokines in the synovial fluid and blood [59,60]. The pathophysiology of rheumatoid arthritis is so complex that its specific mechanism is not yet fully known. However, IL-6 signaling is a significant cause of inflammation and RA symptoms. Cells such as macrophages, osteoclasts, B cells, and T cells have IL-6 and respond to IL-6 signaling, contributing to the immune response in RA. The effectiveness of cytotoxic drugs used in RA therapy is limited by their side effects associated with non-specific targeting. Hence, a formulation strategy is needed to act specifically at the target site. Among the well-known targeted drug delivery strategies, ADCs have recently received much attention for their ability to dispense conjugated drugs to specific cells with negligible adverse drug reactions [61]. 

Lee et al. [26] investigated the effectiveness of tocilizumab–alendronate (TCZ–ALD) conjugate in vitro and in vivo models against RA. TCZ, a humanized mAb, and a small molecule, alendronate (ALD), show anti-inflammatory activity by blocking IL-6 receptors and macrophages, respectively. ALD was attached to the Fc section of TCZ in the presence of a chemical linker via cleavable disulfide bonds without affecting its antigen potential. Conjugation was confirmed by Purpald assay and mass spectrometry. No significant difference in the binding ability of TCZ was observed from the ELISA analysis using TCZ alone and TCZ in the conjugate, confirming that there was no change in antigen binding sites after the conjugation of TCZ. The viability analysis on macrophage RAW 264.7 cells showed reduced viability in the cells treated with ALD compared to the saline, IgG–ALD, and TCZ groups. No significant difference in the cell viability within the treatment groups of the TCZ+ALD mixture and the TCZ–ALD conjugation confirms that there was no alteration in the ALD activity after conjugation.

After in vitro studies, the conjugate activity was evaluated in vivo using collagen-induced arthritis DBA/1j mice. After four weeks of immunization with adjuvant, animals were treated with saline, ALD (0.2 μg/kg), TCZ (0.8 mg/kg) alone, and ALD–TCZ conjugate into the arthritis-induced ankle. Then. animals were observed for the severity of RA and bone degradation for 8 weeks using micro-CT, histological, and Western blot analysis. The micro-CT reveals significant bone degradation in all the animal groups except the TCZ–ALD conjugate-administered group, even though it was given at low doses. This may be due to the synergistic effect of TCZ and ALD on the bone resorption of osteoclasts and the intracellular delivery of ALD due to conjugation with TCZ.

Histological examination indicated the presence of inflammation with the destruction of cartilage in the animals that received ALD alone. This has been significantly reduced in the animals treated with TCZ, whereas the animals treated with TCZ–ALD conjugate showed a clean cartilage-bone contact without inflammatory cell infiltration, nearly like the standard control group. The effectiveness of the TCZ-ALD conjugate was further confirmed by a significant reduction in IL-6 and CD68 levels compared to other groups. The authors concluded that conjugating ALD with TCZ, which specifically binds to IL-6 receptors, extended the residence time of ALD in the joints and showed enhanced efficacy. The success of this conjugate may pave the way for developing more conjugates in autoimmune disorder treatment.

### 2.6. Immuno-Suppression

Dasatinib was clinically used for treating BCR-ABL-dependent chronic myelogenous leukemia. It also has potential immunosuppressive activity due to the inhibition of Src-family kinases such as Lck and Fyn. However, due to a lack of selectivity, dasatinib has severe side effects that limit its clinical application as an immunosuppressing agent. Wang et al. [21] developed an antibody–drug conjugate for the site-specific delivery of dasatinib to T lymphocytes for effective immunosuppression. The authors initially synthesized an antibody specifically targeting T cells and conjugated the antibody to dasatinib in the next step. Finally, the antibody–dasatinib conjugate was evaluated in vitro for immunosuppression activity. Initial screening was performed on several antibodies that could bind to T cells, and CD184 (CXCR4) was selected based on its high expression, better internalization, and fewer side effects. A CXCR4 antagonist was joined with a bovine antibody (BLV1H12) scaffold and further grafted into the CDR3H of trastuzumab to produce fewer immunogenic-humanized antibodies (HLCX). HEK 293F cells were used for the expression of HLCX. Further protein G chromatography was used to purify the antibody, and its mass was confirmed by electrospray ionization mass spectrometry.

A shift in the peak in flow cytometry analysis confirmed the binding of HLCX to CXCR4. The antibody was incubated with cell lines greatly expressing CXCR4 (Jurkat T cells) and cell lines lacking CXCR4 (MDA-MB435 cells). The samples incubated with Jurkat cells clearly showed a shift in the peak (96.2%) due to binding, whereas samples incubated with MDA-MB435 cells did not show any marked shift in the peak due to the absence of binding. This was further confirmed by analyzing the samples incubated with different cell lines with varying levels of CXCR4 expression. The internalization ability of the HLCX into T cells was confirmed by confocal microscopy. Alexa Fluor 488 (AF488) dye was conjugated to the HLCX and an unconjugated antibody and incubated with human T lymphocytes. Green spots were observed in the cytoplasm of T cells within 30 min after mixing at 37 °C, indicative of HLCX-AF488 internalization. This was not observed at 4 °C and with unconjugated HLCX, confirming the site-specific delivery of HLCX. Two ADCs, namely, non-cleavable (HLCX-dasatinib) and cleavable linkers (HLCX-SS-dasatinib), were synthesized in a two-step coupling process after modifying the dasatinib and antibody as both do not have interacting groups directly. Synthesized ADCs were >90% pure, and the drug-to-antibody ratio was 3, as confirmed by SDS phase analysis. The molecular weights were in the anticipated range, suggesting the structural integrity of the synthesized ADCs.

Two ADCs were incubated with activated T cells to produce more CD25 and pro-inflammatory cytokines, including IL-2, TNF-alpha, and interferons (IFN gamma). Two ADCs and unconjugated HLCX were incubated with human T cells that had been activated with either anti-CD3 or anti-CD28 antibodies, and the levels of pro-inflammatory cytokines were analyzed by flow cytometry and ELISA. Flow cytometry and ELISA analysis revealed that both ADCs blocked CD69 and CD25 expression and stopped the release of cytokines at low concentrations (8 nM). In contrast, a minimal effect was observed with the unconjugated HLCX antibody. HLCX-SS-dasatinib was two times more effective than HLCX-dasatinib in suppressing cytokines. This may be due to drug release, internalization, or residence time differences. Both dasatinib and ADCs, at doses up to 200 nM, did not significantly reduce the viability of activated T cells compared to untreated cells measured by CellTiter Glo. This rules out the possibility of cytotoxicity. Western blotting was employed to look at the phosphorylation of downstream kinases during TCR-induced T cell activation. The studies indicated both ADCs and dasatinib block Lck signaling compared to unconjugated antibodies and trastuzumab-SS-dasatinib. This clearly shows that T cell suppression depends on antibodies and drug molecules. Based on the results, it was suggested that ADCs could be a valuable strategy to improve the activity of kinase inhibitors beyond oncological applications.

### 2.7. Atherosclerosis

Atherosclerosis is a condition developed due to the deposition of cholesterol in arteries, responsible for about 35% of deaths in the United States. The process involves the development of oxidized low-density lipoproteins (oxLDL) and a consequent inflammatory response and accumulation of macrophages. The athero-protective effect of macrophages is exerted by transferring cholesterol to the liver for bile secretion through reverse cholesterol transport (RCT). The RCT is triggered by liver X receptors (LXR-α and LXR-β) and others. LXR activation blocks macrophage proliferation and foam cell formation by suppressing pro-inflammatory cytokines. LXR agonists (GW3965 and T0901317) were developed and tested for their activity against atherosclerosis. However, these derivatives also showed increased triglycerides in the liver due to an overexpression of LXR-α. The site-specific delivery of LXR agonists to the macrophages may reduce this risk and improve the clinical applicability of LXR agonists. The ADC-based delivery of LXR agonists to macrophages was described by Lim et al. [22]. The authors synthesized a new compound similar to the above-mentioned LXR agonists that can retain its antigenic nature after linking to an antibody with the substitution of aminoethyl sulfonamide. The protease cleavable linker was found to be suitable after screening the various linkers with disulfides, acid-labile hydrazones, and protease cleavable linkers. Using the chemistry approaches, they synthesized a stable aminooxy-CatB-LXR agonist and confirmed its cleavable potential in the presence of purified CatB enzyme using liquid chromatography and mass spectrometry (LC-MS). The authors selected CD11a as a particular antigen expressed on leukocytes, monocytes, and macrophages but not on the liver. After cloning the humanized antibody Efalizumab and transfecting the Chinese Hamster Ovary (CHO) suspension cells, the required antibody for conjugation, anti-CD11a IgG, was obtained. This was purified, and mass analysis was performed by SDS-PAGE analysis and electrospray-ionization mass spectrometry, respectively. In further experiments, two more antibodies, anti-Her2 IgGX and anti-Her2 FabX, were also expressed as negative controls. Finally, the linker attached to the LXR agonist was conjugated with anti-CD11a IgGX via covalent bonds.

The binding of the conjugate was compared with that of the negative control after incubation with human THP-1 monocyte/macrophage cells and human HepG2 hepatoma cells. Further Fc blockers were also used to confirm that the binding is CD11a-mediated. The flow cytometry analysis indicated anti-CD11a IgGX-AF488 binds to the monophages and macrophages as characterized by distinct peak shifts in flow cytometry analysis. It does not bind to the Hepatoma cells, as indicated by the absence of peak shift. Similarly, the binding of anti-CD11a IgGX-AF488 to monophages exists in the presence of Fc blockers, indicating the binding is CD11a-mediated and not Fc-controlled. Negative control antibody conjugates showed binding in the absence of Fc blockers, and the presence of blockers did not confirm the binding to the cells as they are Fc-dependent. The internalization of the conjugate was established from the identification of the dye (Alexa Fluor 488)-attached conjugate in the cytoplasm of the THP-1 cells after incubation, as observed under confocal microscopy. 

The LXR Transactivation assay was used to examine the LXR agonistic activity of synthesized ADC and compared with that of positive control and T0901317 (LXR agonist) and aminooxy-CatB-LXR agonist. All three compounds were incubated with THP-1 cells and HEpG2 cells in the presence and absence of 10% human AB serum to block Fcγ receptors. Then, the cells were treated with ONE-Glo, and the luminescence was recorded on an Envision plate reader. The synthesized conjugate (anti-CD11a IgGX-AF488) demonstrated a strong agonistic action in THP-1 cells without any activity in HEPG2 cells. The positive control showed equivalent activity in both cell lines, and the aminooxy-CatB-LXR agonist showed no agonistic activity. This indicates that anti-CD11a IgGX-AF488 has a specific binding ability towards macrophages with three-fold higher potency.

### 2.8. Systemic Sclerosis

Systemic sclerosis (SSc) is an autoimmune disorder characterized by immune system dysregulation that causes fibrosis in the skin and viscera. There is no treatment for this disease at present, but treating the patients during the early stages to reverse the inflammation may provide relief. Recent studies revealed that there was a significant enhancement in the inflammatory markers, such as CD28-CD80/86, CD19, CCL24, CD20, CD30, tumor necrosis factor, transforming growth factor β, B-cell activating factor, lysophosphatidic acid receptor 1, soluble guanylate cyclase, Janus kinases, and interleukin 6 in the serum of SSc patients [62]. The enhanced CD30 can be targeted using Brentuximab (αCD30 antibody). Based on the concept, three clinical trials were initiated (clinicaltrials.gov accessed on 19 June 2023, identifierNCT03222492, NCT03198689, NCT05149768). The NCT05149768 trial is a dose-escalation safety study of brentuximab vedotin, a drug–antibody conjugate approved for lymphoma treatment and targeted to the protein CD30 molecule expressed in activated immune cells. There is evidence that CD30 plays a role in SSc. This study is the initial step in evaluating the safety and tolerability of brentuximab vedotin in SSc patients [63]. NCT03198689 is designed to evaluate the feasibility, safety, and preliminary efficacy of Brentuximab vedotin (Adcetris) [64]. The NCT05149768 trial is intended as a 48-week extension of the current therapy to make the treatment available for SSc patients who improved considerably with Brentuximab vedotin (24 weeks treatment) but relapsed after treatment termination [65]. All three trails are in phase II and currently active. According to clinical trial data, the first two studies are expected to be completed by 2023 and the third by 2024.

A clinical trial was proposed on clinicaltrials.gov, accessed on 19 June 2023 (identifier NCT01616680) for using Adcetris in Steroid-Resistant Acute Graft-Versus-Host Disease but it was withdrawn before recruiting the subjects [66]. 

## 3. Clinical Pharmacology Considerations for Antibody–Drug Conjugates

On 20 February 2022, the US Food and Drug Administration (FDA) issued a draft guidance on clinical pharmacology considerations for developing antibody–drug conjugates (ADC). This guidance specifies recommendations to aid the industry and other parties involved in the development of ADCs with an array of payloads. This advice reports the FDA’s current thinking on clinical pharmacology challenges and proposals for ADC development programs, including Bioanalytical Approach, Dose and Exposure-Response, Intrinsic Factors, Pharmacogenomics, and QTc Assessment. These studies must be planned either concurrently with the clinical studies or separately to ensure safety and efficacy. The US FDA’s respective guidance documents can be utilized as a reference to carry out the study. As stated in the guidance, bioanalytical quantification and dose exposure studies are required to examine the safety and efficacy of ADCs and their constituents, as well as any active metabolites. The impact of intrinsic variables on the pharmacokinetics of ADCs must be investigated using organ impairment and pharmacogenomic procedures. Similarly, electrocardiogram (ECG) monitoring should be undertaken for QTc assessment, immunogenicity should be examined to check safety and drug–drug interactions should be investigated to better understand the effect of ADCs in patients who are taking numerous medications [67].

## 4. Conclusions and Perspectives

The current review summarized the various studies that explored the prospect of extending the utility of ADCs in non-oncological disease conditions such as inflammation, infectious diseases, systemic sclerosis, atherosclerosis, and immunosuppression. Even though ADCs gained much attention in the oncology treatment due to their site-specific delivery and reduced off target effects, the use of ADCs in non-oncology condition was limited to specific payloads that need site-specific delivery to reduce the side effects. In such circumstances, the benefits of ADCs for such payloads should be weighed against the benefits of therapy, the cost of therapy, and alternative options for such payloads. The studies also need to focus on the regulatory aspects and consider the various studies that need to be performed for fulfilling the regulatory requirements. Another issue to be concerned about is the use of antibodies that are specific to the overexpressed antigens. Many research investigations investigated the issue of producing antibodies in non-oncological illnesses. Future research should consider the concerns raised in order to successfully develop ADCs for the treatment of non-oncological diseases.

## Figures and Tables

**Table 1:** A list of ADCs that have been tested for indications other than oncology.

Indications	ADCs	Antibody	Linkers	Payloads	Testing Status	Ref.
Inflammation	Anti-E Selectin Dex (Dexa–AbhEsel)	Murine anti-E-selectin mAb (H18/7)	Succinate linker	Dexamethasone	In vitro pre-clinical	[17]
Chronic models of inflammation	Anti-CD163 Dex (Cymac-001)	Murine anti-CD163 mAb (Ed-2)	Hemisuccinate linker	Dexamethasone	In vivo pre-clinical	[19]
Autoimmune models	Anti-CD74 fluticasone propionate (Anti-CD74-flu449)	Human anti-CD74 mAb	Pyrophosphate acetal linker	Fluticasone propionate	In vivo pre-clinical	[20]
Autoimmune and inflammatory models	Anti-CXCR4 dasatinib	Humanized anti-CXCR4 mAb (HLCX)	Tetra-poly-ethylene glycol linker	Dasatinib	In vitro pre-clinical	[21]
Atherosclerosis	Anti-CD11a LXR agonist	Humanized anti-CD11 mAb	PEG4-Phe-Lys	Amino acid para-acetylphenylalanine	In vitro pre-clinical	[22]
Muscular diseases	Anti-CD71 siRNA	Murine anti-CD71	mAb Maleimide linker	siRNA	In vivo pre-clinical	[23]
Myasthenia gravis	Anti-TNFRSF13c siRNA	anti-TNFRSF13c mA	Protamine linker	siRNA	In vivo pre-clinical	[24]
Systemic sclerosis	Anti-CD30 Vedotin (ADCETRIS)	Chimeric anti-CD30 mAb (cAC10, SGN-30)	Val-Cit linker	MMAE (Monomethyl auristatin E)	Phase II clinical trial (NCT03198689), (NCT03222492)	[25]
Rheumatoid arthritis	Anti-IL-6 alendronate	Humanized anti-IL-6 mAb (tocilizumab)	PDPH-PEG-NHS	Alendronate (ALD)	In vivo pre-clinical	[26]
Rheumatoid arthritis	Anti-FRβ Pseudomonas exotoxin A (PE38)	Murine anti-FRβ mAb	NA	*Pseudomonas* exotoxin A (PE38)	In vivo pre-clinical	[27]
Rheumatoid arthritis	Anti–C5aR1 C5 siRNA	Murine anti-C5aR1 mAb	Protamine linker	C5 siRNA	In vivo pre-clinical	[28]
*S. aureus* bacteremia	Anti-*S. aureus* Antibiotic (DSTA4637S)	Human anti-β-Nacetylglucosamine cell-wall teichoic acid (β-GlcNAc- WTA) mAb	MC-Val-Cit-PAB-OH	Antibiotic (rifalogue)	Phase I clinical trial (NCT03162250)	[29]
Arthritis	Anti-mTNF GRM (ABBV-3373)	Antibody: alpha TNF	MP-Ala-Ala	Glucocorticoid Receptor Modulator (GRM)	In vitro pre-clinical	[30]
Arthritis	α-TNF-GRM ADC	Antibody: alpha TNF	M-Gly-Ala-Ala	GRM	In vitro pre-clinical	[31]
Inflammation	Anti-TNFα glucocorticoid	Anti-TNF mAb	Dipeptide-based (Ala-Ala) protease-cleavable	Dexamethasone	In vitro pre-clinical	[15]
Non-alcoholic fatty liver disease	Anti-CD163-IgG- Dex	Anti-CD163 mAb	Hemisuccinate linker	Dexamethasone	In vivo pre-clinical	[32]
Bowel disease, ulcerative colitis, and Crohn’s disease.	anti-CD70 mAb–Budesonide	Anti-CD70 mAb	Carbamate linkage	Budesonide	In vitro and vivo pre-clinical study	[33]
Theragnostic agent	Trastuzumab-7- nitro-3-hydroxyethyl- coumarin- Monomethyl auristatin E	Trastuzumab	Carbamate linkage	L-233	In vitro pre-clinical study	[34]
Staphylococcus Aureus infections	THIOMAB™ antibody antibiotic conjugate/ DSTA4637A	Human IgG1 anti-*S. aureus* THIOMAB™ monoclonal antibody	valine-citrulline linker	Antibiotic dmDNA31	In vitro and In vivo study	[35]
Lung infection	VSX-D297 antimicrobial antibody conjugate	VSX	Enzymatically coupling with Sortase A	Antimicrobial peptides	In vitro studies	[36]
Orthopedic implant-associated intracellular *S. aureus* infections	M0662-MC-Val-Cit-PAB-Vancomycin	Human monoclonal antibody (M0662) against the surface antigen Staphylococcal protein A (SpA) of *S. aureus*	Mc-Val-Cit-PAB	Vancomycin	In vitro studies	[37]

## Data Availability

Not applicable.
